# Optimizing Exchange Transfusion for Severe Unconjugated Hyperbilirubinemia: Studies in the Gunn Rat

**DOI:** 10.1371/journal.pone.0077179

**Published:** 2013-10-15

**Authors:** Andrea B. Schreuder, Jana Vanikova, Libor Vitek, Rick Havinga, Charles E. Ahlfors, Christian V. Hulzebos, Henkjan J. Verkade

**Affiliations:** 1 Pediatric Gastroenterology and Hepatology, Department of Pediatrics, Center for Liver, Digestive, and Metabolic Diseases, Beatrix Children’s Hospital - University Medical Center Groningen, University of Groningen, Groningen, The Netherlands; 2 Institute of Medical Biochemistry and Laboratory Diagnostics, 1st Faculty of Medicine, Charles University, Prague 2, Czech Republic; 34 4th Department of Internal Medicine, 1st Faculty of Medicine, Charles University, Prague 2, Czech Republic; 4 Stanford University, School of Medicine, Stanford, California, United States of America; 5 Neonatology, Department of Pediatrics, Beatrix Children’s Hospital - University Medical Center Groningen, Groningen, The Netherlands; University of North Carolina School of Medicine, United States of America

## Abstract

**Background:**

Severe unconjugated hyperbilirubinemia carries the risk of neurotoxicity. Phototherapy (PT) and exchange transfusion (ET) are cornerstones in the treatment of unconjugated hyperbilirubinemia. Studies to improve ET efficacy have been hampered by the low application of ET in humans and by the lack of an *in vivo* model. The absence of an appropriate animal model has also prevented to determine the efficacy of adjunct or alternative treatment options such as albumin (Alb) administration.

**Aim:**

To establish an *in vivo* model for ET and to determine the most effective treatment (combination) of ET, PT and Alb administration.

**Methods:**

Gunn rats received either PT, PT+Alb, ET, ET+PT, ET+PT+Alb or sham operation (each n = 7). ET was performed *via* the right jugular vein in ∼20 min. PT (18 µW/cm^2^/nm) was started after ET or at T_0_. Albumin *i.p.* injections (2.5 g/kg) were given after ET or before starting PT. Plasma unconjugated bilirubin (UCB), plasma free bilirubin (Bf), and brain bilirubin concentrations were determined.

**Results:**

We performed ET in 21 Gunn rats with 100% survival. At T_1_, ET was profoundly more effective in decreasing both UCB −44%, p<0.01) and Bf −81%, p<0.05) than either PT or PT+Alb. After 48 h, the combination of ET+PT+Alb showed the strongest hypobilirubinemic effect (−54% compared to ET).

**Conclusions:**

We optimized ET for severe unconjugated hyperbilirubinemia in the Gunn rat model. Our data indicate that ET is the most effective treatment option, in the acute as well as the follow-up situation.

## Introduction

Neonatal jaundice carries the risk of neurotoxicity, due to the deposition of unconjugated bilirubin (UCB) in the central nervous system. Most of the UCB (∼99%) in plasma is bound to plasma proteins (mainly albumin). Only a small fraction (∼1%) is “free”, and only this free bilirubin (Bf) has the ability to cross the blood-brain barrier and to induce brain damage [1]–[5].

Presently, the standard treatment for hyperbilirubinemia is phototherapy. Phototherapy (PT) is generally effective, but in some neonates the plasma bilirubin concentrations become dangerously high or rise rapidly despite PT. In these patients PT might fail to prevent bilirubin-induced brain damage, and for these patients exchange transfusion (ET) is indicated. Exchange transfusions have more serious side effects and complications than PT. The mortality rate from the procedure is approximately 0.3–2.0%. Significant morbidity is associated with 5–12% of ETs [6]–[8]. Complications include cardiac arrest, thrombosis of the portal vein, graft vs. host disease, coagulopathies, hypoglycemia, hypocalcaemia, necrotizing enterocolitis, and transmission of infectious diseases [6]–[10].

It has remained unclear whether ET could successfully be replaced by other, more effective treatment options. For example, albumin infusion might be a good treatment modality. Recently, we found that adjunct human serum albumin (HSA) increased the efficacy of PT; it decreased plasma Bf concentrations and brain bilirubin levels by ∼90% and ∼70%, respectively [11]. Studies to replace ET, to improve ET efficacy and/or to minimize its risks have been hampered by the contemporary low application rate of ET in humans and by the lack of an appropriate *in vivo* model system. In order to better study the effects of an ET, animal studies would be highly desirable. An appropriate animal model should resemble the human situation as much as possible. In case of ET for hyperbilirubinemia, it should lower the bilirubin levels sufficiently, quickly and safely. In this study we set out to establish an animal model for ET, in which we would be able to evaluate the effect of an ET on bilirubin concentrations in the acute and long-term situation.

We used Gunn rats suffering from hyperbilirubinemia due to a mutation in uridine diphosphoglucuronosyltransferase: UGT1A1 [12]–[15]. The Gunn rat is a well-established animal model for unconjugated hyperbilirubinemia. The histopathological lesions in severely kernicteric Gunn rats include damage to central auditory structures, especially the cochlear nuclei and inferior colliculi, and are similar to those found in human neonates with classic kernicterus [16].

In the present study, we successfully optimized and verified an ET model in Gunn rats to compare acute treatments for severe hyperbilirubinemia. Next, we evaluated different acute treatment options for hyperbilirubinemia with or without the combination of ET, and compared total serum bilirubin, free bilirubin and brain bilirubin levels.

## Animals, Materials, and Methods

### Animals

Homozygous male Gunn rats (RHA/jj; 10–12 weeks of age, bodyweight: 254–335 g) from our breeding colony were kept in an environmentally controlled facility, were fed ad libitum and had free access to water. The Animal Ethics Committee of the University of Groningen (Groningen, The Netherlands) approved all experimental protocols.

### Materials

#### Diet

Hope Farms B.V. (Woerden, The Netherlands) produced the semi-synthetic control diet (code 4063.02). This diet contained 13 energy% fat and 5.2 wt% long-chain fatty acids. Gunn rats were fed this diet during a 5-week run-in period, and during the experimental period.

#### Chemicals

Horseradish peroxidase type 1, D-glucose, glucose oxidase, and hydrogen peroxide were purchased from Sigma Chemical Co. (St. Louis, MO). Human serum albumin (Albuman®; 200 g/L, fatty acid free) was purchased from Sanquin (Amsterdam, The Netherlands).

### Methods

#### Phototherapy

Two phototherapy devices were developed according to the prototype that was designed by Ostrow *et al.* and previously successfully used [11], [17]. Each device consisted of two blue phototherapy lamps (Philips, TL-20W/52) suspended in a reflective canopy 30 cm above the bottom of the cage. Phototherapy (18 µW/cm2/nm; 380–480 nm; measured by an Elvos-LM-1010 Lux meter at 30 cm distance), was administered continuously to Gunn rats, shaven on their backs and flanks.

#### Exchange transfusion

Fresh whole rat Wistar donor blood was obtained from Harlan Laboratories B.V. (Horst, The Netherlands). Exchange transfusion was carried out under general anesthesia with isoflurane. Body temperature was maintained at 37–38°C by a heating plate. Saturation was checked and kept constant during the whole procedure above 95%. Different vessel approaches, including femoral artery and vein, the carotic artery and jugular vein, have been tested and the following description was used for all experiments. A small incision was made in the right throat region and, with the aid of an operating microscope, the right jugular vein was cannulated with heparinized silastic tubing for the infusion of donor blood, and the extraction of the native blood. In total 20 ml of donor blood was infused via a heparinized lock, and 20 ml of native blood was taken out (1 ml per cycle in 1 minute). Exchange transfusion was performed at a rate of 1 ml/min, for 20 minutes. Blood outflow was performed by hand using 1 ml syringes, and donor blood inflow was performed using an infusion pump. After the exchange transfusion tubes were ligated and left in situ, and the skin was sutured.

#### Sham transfusion

Sham transfusion was carried out following the same procedure as the exchange transfusion. After cannulation of the jugular vein, animals were kept under general anesthesia for 20 minutes. After the sham the heparinized silastic tubings were ligated extra corporally and the proximal part was left in the jugular vein *in situ*. Finally, the skin was sutured.

### Study Design

Adult Gunn rats were randomized to receive either sham operation without treatment (controls), phototherapy, phototherapy+HSA, an exchange transfusion, an exchange transfusion+phototherapy or an exchange transfusion+HSA+phototherapy (each of these groups n = 7). The exchange transfusion (ET) group underwent ET at a rate of 1 ml/min for 20 minutes. HSA *i.p.* injection (2.5 g/kg) was given immediately after the ET or right before PT was started. Heparinized samples of tail vein blood were collected, under isoflurane anesthesia, at time (t) = 0 (before the ET), at t = 1, t = 3, t = 6, and t = 24 h after the ET. After 48 h, all animals were exsanguinated via the descending aorta and flushed via the same port with 100–150 ml NaCl 0.9% under isoflurane anesthesia. Brains were subsequently collected for the determination of tissue bilirubin levels. These samples were rinsed twice with phosphate buffered saline, snap frozen in liquid nitrogen, and immediately stored (wrapped in aluminum foil) at −80°C until analysis.

### Analytical Methods

#### Plasma analysis

Blood samples were protected from light, stored at −20°C under argon directly after collection and processed within 2 weeks. UCB and Bf were determined using a Zone Fluidics system (Global Flopro, Global Fia Inc, WA), as previously described by Ahlfors *et al.* [18].

#### Tissue bilirubin analysis

Tissue bilirubin content was determined using HPLC with diode array detector (Agilent, Santa Clara, CA) as described earlier [19]. Briefly, 300 pmol of mesobilirubin in DMSO (used as an internal standard) was added and samples were homogenized with glass dust using glass rod. Bile pigments were then extracted into chloroform/methanol/hexane (10∶5∶1) solution at pH 6.0, and subsequently extracted in a minimum volume of methanol/carbonate buffer (pH 10) to remove contaminants. The resulting polar droplet (extract) was loaded onto C-8 reverse phase column (Phenomenex, Torrance, CA) and separated pigments were detected at 440 nm. The concentration of bilirubin was calculated as nmol/g of wet tissue weight. All steps were performed under dim light in aluminum-wrapped tubes. We did not specifically measure bilirubin deposition in the brain nuclei, but relied on total tissue bilirubin measurements.

#### Statistical analysis

Normally distributed data that displayed homogeneity of variance (by calculation of Levene’s statistic) were expressed as mean ± SD, and analyzed with parametric statistical tests. Analysis of variance (ANOVA) with post-hoc Tukey correction was performed for comparisons between groups, and the Student *t* test for comparison of paired data within groups. The level of significance was set at p<0.05. Analyses were performed using SPSS Statistics 20.0 for Windows (SPSS Inc., Chicago, IL).

## Results

### Development and Validation of the Model

In the human situation the common route for ET involves catheterization of the umbilical vein, and arteriovenous or venovenous exchange. Initially, we set out to establish arteriovenous exchange *via* the femoral artery and vein. Based on the anatomical location, the femoral artery was not suitable for exchange procedure. We then switched first to arteriovenous exchange *via* the carotid artery and jugular vein, but this method failed because of the high pressure in the carotid artery, which made it impossible to keep the cannula in place for more than 5 minutes. Finally, we moved on to venovenous exchange *via* the jugular vein on both sides. When we found out that we received the same results in decrease of plasma bilirubin concentrations *via* the push-and-pull-method *via* one jugular vein, as *via* the continuous exchange *via* both jugular veins, we decided to continue with the venovenous exchange *via* one jugular vein. The rats recovered quickly with this method. *Via* the same jugular vein we infused fresh Wistar donor blood (UCB <1 mg/dL), and extracted the blood of the Gunn rat, in 20 minutes. We tested different lengths of the procedure and observed that 20 minutes procedure led to the same decrease of plasma UCB concentrations as 40 and 60 minutes procedures (same volume, data not shown).

**Figure 1:**
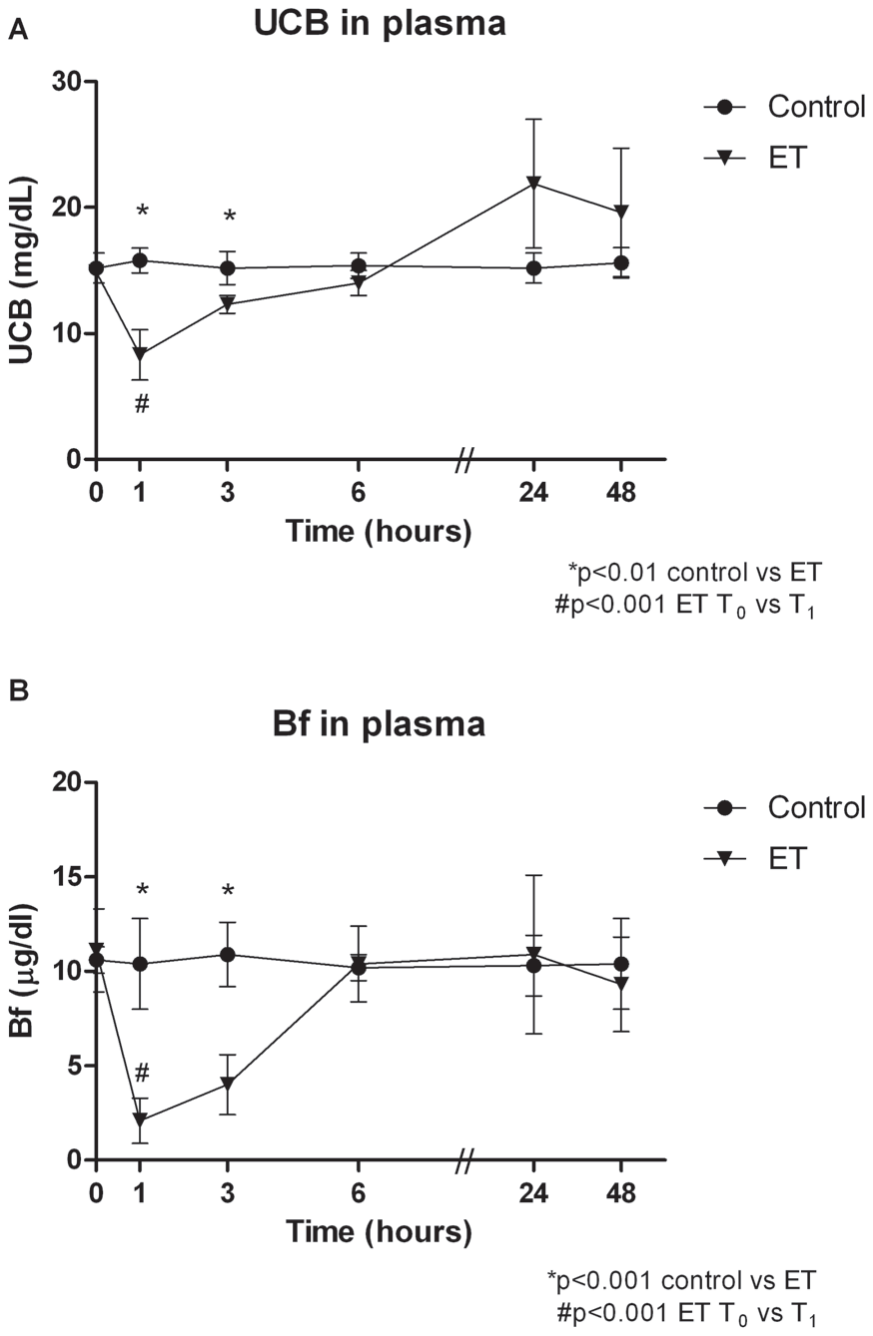
Course of plasma UCB and Bf concentrations after exchange transfusion. Course of plasma UCB concentrations (**A**) and plasma Bf concentrations (**B**) after sham transfusions (control) or an exchange transfusion (ET) in Gunn rats. Rats were randomized to receive sham transfusions (control) or an exchange transfusion (ET). Values are mean ± SD. *p<0.01 compared to controls. #p<0.001 ET: T_0_ compared to T_1_.

We performed an ET in 21 Gunn rats with 100% survival. The recovery after ET was rapid, illustrated by maintenance of body weight during the 48 h after ET (at T_48_, 100±3% compared to T_0_, NS). Figure 1A shows the course of plasma UCB concentrations after ET. ET rapidly decreased plasma UCB concentrations from 14.9 mg/dL at T_0_, to 8.3 mg/dL at T_1_ (−44%, p<0.001). In Figure 1B the course of plasma Bf concentrations after ET is shown. ET decreased plasma Bf concentrations from 11.1 µg/dL at T_0_, to 2.1 µg/dL at T_1_ (−81%, p<0.001). At T_6_, T_24_ and T_48_ no significant difference exist in plasma Bf concentrations between controls and ET.

### Plasma UCB Concentrations after 1 h

**Figure 2:**
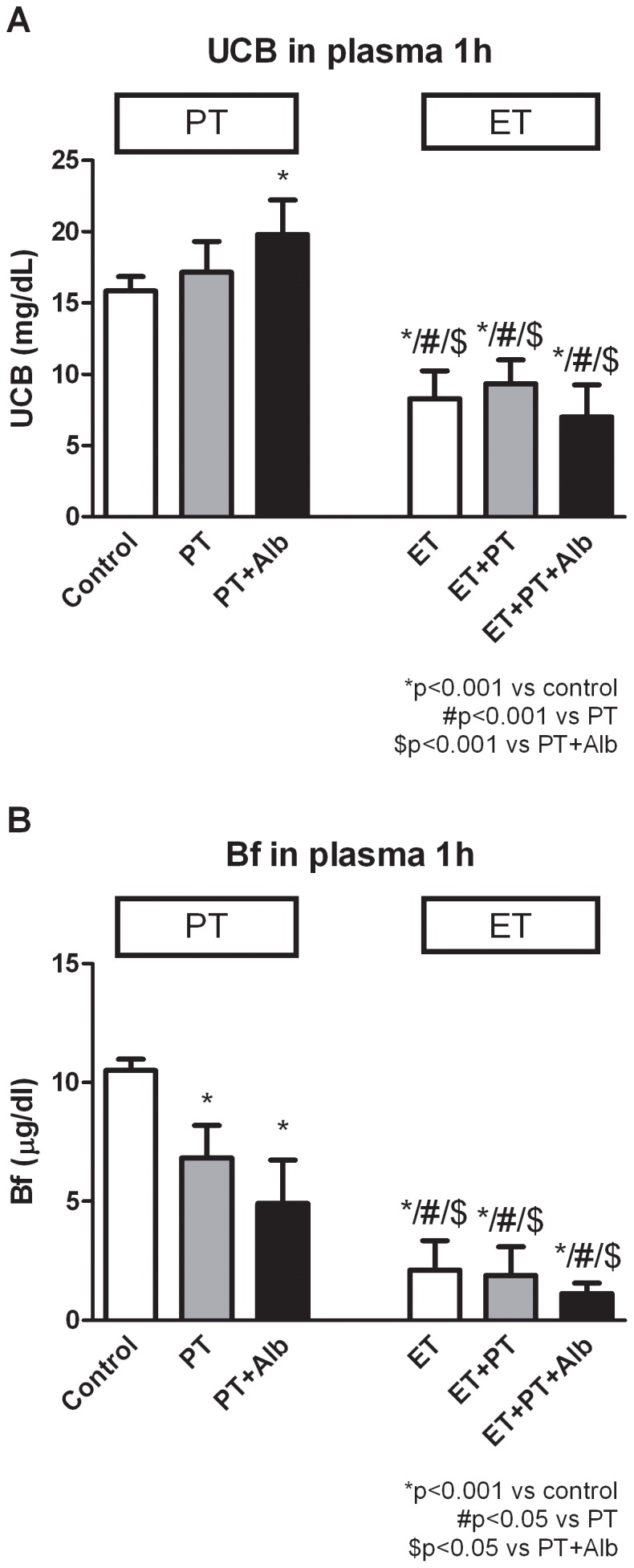
Plasma UCB and Bf concentrations after 1 h. Acute effects of sham transfusions (control) or phototherapy (PT), albumin (Alb), an exchange transfusion (ET), or a combination of these on plasma UCB concentrations (**A**) and plasma Bf concentrations (**B**) in Gunn rats. Rats were randomized to receive sham transfusions (control) or an exchange transfusion (ET), and were subsequently treated with phototherapy (PT), albumin (Alb) or the combination of PT+Alb. Values are mean ± SD. *p<0.001 compared to controls. #p<0.05 compared to PT. $p<0.05 compared to PT+Alb.

We compared the acute effect of the different treatments; PT, ET, Alb administration or a combination thereof (Figure 2A). After 1 h, PT showed no significant differences in plasma UCB concentrations compared to controls. PT+Alb showed a significant increase compared to controls (p<0.001). In contrast, ET reduced plasma UCB concentrations by 47% within 1 h (p<0.001 vs controls). The addition of either PT or the combination of PT and Alb did not significantly augment this hypobilirubinemic effect. Each of the combination therapies that included ET resulted in a significantly lower plasma UCB concentration compared to the control, PT or PT+Alb groups (each p<0.001).

### Plasma Bf Concentrations after 1 h

Figure 2B shows the effects of the different treatment combinations on plasma Bf concentrations. After 1 h, PT and PT+Alb reduced plasma Bf concentrations with 35% (p<0.001 vs controls) and 53% (p<0.001 vs controls), respectively. For the ET-group, ET+PT-group and ET+PT+Alb-group the decrease in plasma Bf concentrations was even more profound (−80%, −80% and −89% respectively; each p<0.001 vs controls, no statistically significant difference between the three ET groups). Also, the different ET-groups each showed significantly lower plasma Bf concentrations compared to PT+Alb (p<0.05).

### Plasma UCB Concentrations after 48 h

**Figure 3:**
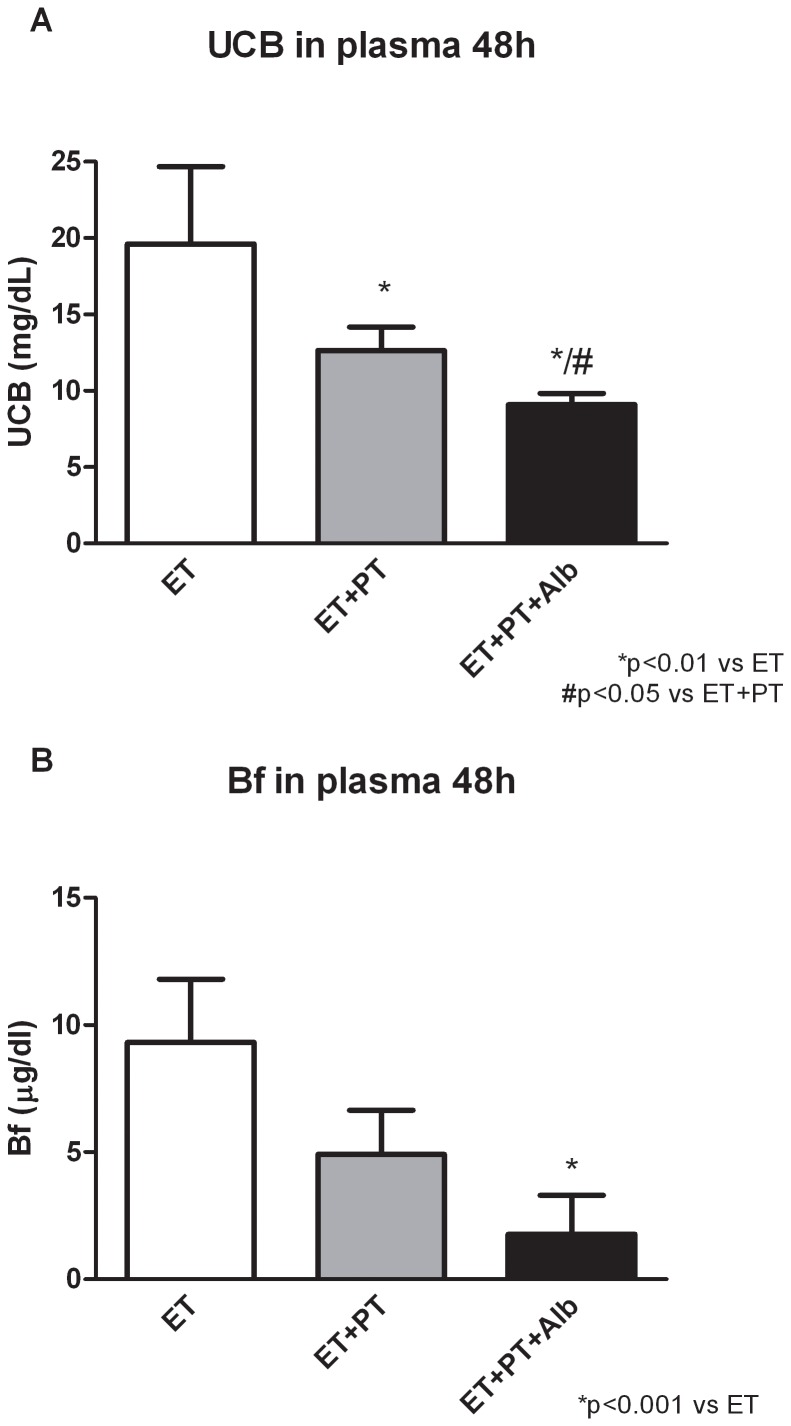
Plasma UCB and Bf concentrations after 48 h. Long-term effects of an exchange transfusion (ET), with or without the combination of phototherapy (PT), or albumin (Alb), on plasma UCB concentrations (**A**) and plasma Bf concentrations (**B**) in Gunn rats. Rats were randomized to receive an exchange transfusion (ET), and were subsequently treated with phototherapy (PT), albumin (Alb) or the combination of PT+Alb. Values are mean ± SD. *p<0.01 compared to ET. #p<0.05 compared to ET+PT.

We also determined the long-term (48 h) hypobilirubinemic effect of the different treatments. We compared treatment combinations that are also used in the clinical practice: an ET with or without the combination of PT or Alb administration. Figure 3A shows the effects of the different treatment combinations on plasma UCB concentrations after 48 h. In the ET-group the plasma UCB concentrations returned back to physiological Gunn rat values as described in the validation of the model. ET+PT significantly reduced plasma UCB concentrations compared to ET after 48 h (−36%; p<0.05). Albumin further potentiated this effect, shown in the significant decrease of ET+PT+Alb compared to ET+PT (−28%; p<0.05).

### Plasma Bf Concentrations after 48 h

Figure 3B shows the effects of the different treatment combinations on plasma Bf concentrations. After 48 h, the plasma Bf concentrations of the ET was still significantly lower compared to controls (−48%; p<0.05 vs controls; data not shown). ET+PT further reduced plasma Bf concentrations with 47% (NS, vs ET). Albumin potentiated the decrease in plasma Bf concentrations after 48 h, shown in a profound, significant decrease of ET+PT+Alb compared to ET (−81%; p<0.01).

### Brain UCB Levels

**Figure 4:**
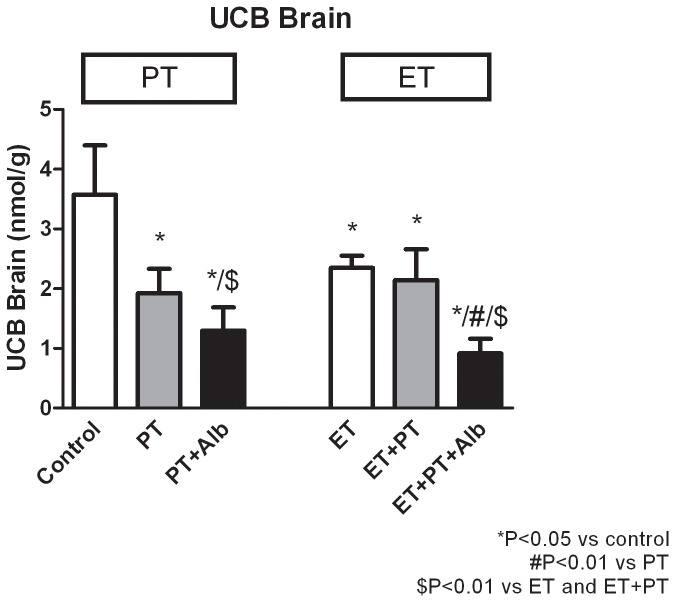
Brain bilirubin levels. Effects of sham transfusion (controls), phototherapy (PT), albumin (Alb), an exchange transfusion (ET) or a combination ofn these on brain bilirubin levels in Gunn rats. For experimental setup, kindly refer to the Methods section. Values are mean ± SD. *p<0.05 compared to controls. #p<0.01 compared to PT. $p<0.05 compared to ET and ET+PT.

Figure 4 shows that PT+Alb decreased brain bilirubin levels by 63% (p<0.001), compared with untreated controls. Adjunct albumin thus lowered brain bilirubin levels by an additional 33% (NS), compared with phototherapy alone. ET+PT+Alb decreased brain bilirubin levels by 61% (p<0.01), compared with ET. Adjunct albumin thus lowered brain bilirubin levels by an additional 57% (p<0.01), compared with ET+PT.

## Discussion

In this study we successfully optimized ET during unconjugated hyperbilirubinemia in a Gunn rat model. We also bring the evidence that this Gunn rat-ET model might be very valuable to evaluate the effect of modulating ET procedures and techniques, and to compare its efficacy in combination with other treatments to prevent brain damage during acute severe hyperbilirubinemia. Our data indicate that ET is highly effective in decreasing UCB and Bf within 1 h of treatment, and that combining ET with either PT or PT+Alb does not further significantly potentiate this rapid hypobilirubinemic effect. As follow up treatment after ET, the combination of PT with Alb is most effective in maintaining this hypobilirubinemic effect over 48 h.

Presently, ET is a very effective alternative treatment to PT in severely jaundiced neonates. An ET is considered as a “rescue treatment”, if plasma UCB levels are severely elevated or fail to respond to PT. Exchange transfusion generally reduces plasma UCB concentrations by 50%, although the efficacy varies with the severity of the ongoing hemolysis and the amount of bilirubin that re-enters the circulation from the tissues [20]. This re-entry occurs due to the diffusion of Bf from the tissue pool into the plasma pool and decreases the risk of bilirubin-induced neurotoxicity [20]. Eventually, all therapy should aim to prevent neurotoxicity, and this can only be achieved by decreasing (brain) tissue rather than plasma UCB concentrations. Nevertheless, an ET has a considerable morbidity, and even mortality has been reported [6]–[8]. Fortunately, the need for ETs has been greatly reduced since the introduction of PT [21], [22].

Our model makes it possible to determine if we can replace ET, improve its efficacy and/or minimize its risks. An alternative treatment option would be the administration of human serum albumin (HSA). HSA-infusion can be used in combination with an exchange transfusion in severely jaundiced neonates, when donor blood is not immediately available [23], but this approach has been disputed [23], [24]. The rationale for HSA-infusion is that the resultant increase in albumin concentration will enhance the bilirubin/albumin-binding capacity in the intravascular compartment, thereby promoting the mobilization of bilirubin from extravascular tissues, including the central nervous system, into the circulation. In this way albumin is used as an adjunct treatment in order to more efficiently remove bilirubin [23].

Our experimental design on albumin administration differs to clinical ET practices in humans with respect to dosage, timing, and route of administration. In the clinics, albumin may be administered to hyperbilirubinemic neonates, but its use seems relatively rare. If administered, it has been advised to do so prior to ET, aimed to increasing its efficacy by mobilizing bilirubin from tissues. In the present study, we administered albumin immediately after ET, aimed at preventing or mitigating a possible rebound of Bf after ET. We administered albumin in a relatively high dosage (2.5 g/kg, rather than ∼1 g/kg in humans) *via* intraperitoneal bolus injection, in contrast to *i.v.* infusion in humans. It should be underlined that these methodological differences prevent direct extrapolation of our present result towards the clinical situation. Rather, present positive “proof of principle” results in our rat model support the design of clinical studies in this direction.

Recently, we found that HSA enhances the efficacy of routine PT in phototherapy-treated Gunn rats, both during permanent and acute jaundice [11]. We speculated that HSA and PT work *in tandem*: HSA binds bilirubin within the plasma, and PT then promotes its excretion *via* the bile [11]. In this study we showed that albumin administration in combination with either PT or ET is already effective after 1 h of treatment. Furthermore, we showed that the combination of PT+Alb is effective in decreasing plasma UCB concentrations, plasma Bf concentrations and brain UCB levels after 48 h. However, in this study we found that ET decreases UCB and Bf concentrations even more than PT+Alb, both in the acute and the chronic treatment situation. Our data demonstrate that ET is still the most effective treatment option in acute severe hyperbilirubinemia. Unfortunately, we were not able to measure plasma albumin concentrations. Albumin is believed to exert its beneficial effects in the blood circulation. In a previous study in adult Gunn rats, we showed that HSA readily enters the plasma compartment after *i.p.* injections [11]. It is worth mentioning that in this previous study we have applied “albumin only” treatment, *i.e.* without prior ET. “Albumin only” decreases the plasma Bf with 54% after 48 hr [11]. The present results justify a follow-up study on an ET+Alb-group. However, in the present study-design we focused on the comparability of our animal-experiments with the clinical situation. Next, it is worth mentioning that in adult rats, PT may not be as efficient as in rat pups. Both skin thickness and body mass/surface ratio are increased in adults, thus underestimating the potential of PT together with ET.

In our ET-model we chose a venovenous exchange *via* one jugular vein in 20 minutes. Various models for exchange of blood are described, each for different purposes. Eguchi *et al.* performed a total blood exchange in rats, and showed that total blood exchange suppressed the early stage of liver regeneration following partial hepatectomy [25]. These scientists performed the total blood exchange *via* the right femoral vein and artery. Henry *et al.* improved monoclonal antibody tumor/background ratios with ETs in rats [26]. They used the right common carotid artery for the blood exchange. Takeda *et al.* studied the effect of blood ET as an initial treatment of acute hemorrhagic pancreatitis in rats [27]. Blood ET was performed *via* a previously indwelt tube in the inferior vena cava. Hodges *et al.* studied the effect of an ET on the efficacy of penicillin therapy of pneumococcal infection in rats [28]. The left external jugular vein was used to perform the ET. We based our model to a certain extent on the latter approach. Kurantsin-Mills *et al.* studied flow dynamics of human sickle erythrocytes in the mesenteric microcirculation of rats that underwent an ET via the femoral vein [29]. The time schedule we used for infusion and blood outflow was partly based on this study. Since we had a different goal than the studies described above, namely exchange of hyperbilirubinemic blood, we decided to develop our own model.

We used fresh donor-rat blood, collected at the same day as the ET takes place. The life span of erythrocytes is approximately 120 days in human adults, 90 days in neonates, and 50–60 days in rats [30]. However, the storage time of red blood cells for rats is much shorter than for human red blood cells, maximum 7 days compared to maximum 30 days respectively [31]. Hemolysis of rat red blood cells happens quickly, and after 7 days all the red blood cells are lysed [31].

In conlusion, we successfully optimized and verified an animal model for ET for treatment of severe unconjugated hyperbilirubinemia. Our data indicate that ET is a more effective treatment option for acute hyperbilirubinemia, than either PT or the combination of PT and Alb. The combination of PT and Alb was the most effective follow up treatment after ET for long term (48 h) hypobilirubinemic effect. The availability of this optimized model could be very helpful to further optimize the treatment for acute, potentially neutotoxic hyperbilirubinemia.
